# Comparative survey study of the use of a low cost exoscope vs. microscope for anterior cervical discectomy and fusion (ACDF)

**DOI:** 10.3389/fmedt.2022.1055189

**Published:** 2023-01-04

**Authors:** Manuel Encarnacion Ramirez, Ismael Peralta Baez, Renat Nurmukhametov, Efgeni Goncharov, Ibrahim E. Efe, Albert Sufianov, Issael Ramirez Pena

**Affiliations:** ^1^ Department of Neurosurgery, RUDN University, Moscow, Russia; ^2^ Department of Neurosurgery, Hospital Regional Alejandro Cabral, San Juan de la Maguana, Dominican Republic; ^3^ Traumatology and Orthopedics Center, Central Clinical Hospital of the Russian Academy of Sciences, Moscow, Russia; ^4^ Department of Neurosurgery, Charité – Universitätsmedizin Berlin, Corporate Member of Freie Universität Berlin, Humboldt-Universität zu Berlin, and Berlin Institute of Health, Berlin, Germany; ^5^ Department of Neurosurgery, First Moscow State Medical University (Sechenov University), Moscow, Russia; ^6^ Department of Neurosurgery, The Royal Melbourne Hospital, Melbourne, VIC, Australia

**Keywords:** cervical disc disease, anterior cervical discectomy, anterior cervical fusion, exoscope, low cost exoscope

## Abstract

**Background:**

Anterior cervical discectomy and fusion (ACDF) is an often performed procedure in spine neurosurgery. These are often performed using an operating microscope (OM) for better illumination and visualization. But its use is limited to the surgeon and the assistant. There is difficulty in maneuvering long surgical instruments due to the limited space available. Exoscope (EX) has been used as an alternative to microscopes and endoscopes. We used an EX in patients undergoing ACDF for cervical spondylotic myelopathy.

**Methods:**

A prospective comparative trial was conducted to test the safety and usability of a low-cost EX compared to a conventional surgical binocular OM in ACDF. Twenty-six patients with degenerative cervical myelopathy symptoms were operated by ACDF assisted by the EX and OM between December 2021 and June 2022. The authors collected and compared data on operative time, intraoperative hemorrhage, hospital admission, and complications in the two groups.

**Results:**

There were no statistically significant differences between the two groups in mean operative time, hospital stay, or postoperative complications. The average intraoperative blood loss was significantly more in the OM group. There were no surgical complications related to the use of the EX or OM. The comfort level, preoperative setup and intraoperative adjustment of position and angle of the EX were rated higher than the OM group. The image quality, depth perception, and illumination were rated as inferior to that of the OM. The low-cost EX was rated to be superior to that of the OM with regard to education and training purposes.

**Conclusion:**

Our study showed that the low-cost EX appears to be a safe and effective alternative for OM-assisted ACDF with great comfort and ergonomics and serves as an essential tool for education and training purposes. However, some limitations of our EX included slightly inferior image quality and illumination when compared with the OM.

## Introduction

Anterior cervical discectomy and fusion (ACDF) is considered the standard treatment of disc herniation in the context of degenerative cervical myelopathy (DCM) causing anterior neural compression (1, 2). It is frequently performed in spine neurosurgery (1–4). These are often performed using an operating microscope for improved illumination and visualization of underlying structures (5). The operating microscope (OM) allows for a safer and more effective surgery (2, 6). But its use is limited to the surgeon and the assistant. There is difficulty in maneuvering long surgical instruments because of the limited space available (3).

Since the introduction of exoscope (EX), it has been used as an alternative to microscopes and endoscopes (2, 3, 5, 7). The main advantage of 3D EX-assisted surgery is the feasibility to generate high quality videos and images which can be perceived equally by the surgeons, assistants and operating room staff (3, 8), in addition, other advantage is the compact size of the EX compared with OM, facilitating free maneuverability and comfort from the onset of surgery (3, 5, 9).

High purchase costs prevent the widespread use of OM and EX in resource-constrained areas. We have previously described a more cost-friendly and simple EX constructed of industrial digital microscope parts with enough capacity for magnification and luminescence during both cranial and spinal surgeries (10), however, zoom and camera maneuvering continued to be limiting factors (10). The lack of stereopsis and the necessity, like any new device to develop hand-eye coordination, have also been previously described as limitations (3, 9, 11, 12).

We used an EX in patients with an ACDF approach for disc herniation in the context of DCM.

## Methods

A prospective comparative trial was conducted to test the safety and usability of a self made low-cost EX compared to a conventional OM in ACDF. The study was performed in the Department of Spine Surgery at the Hospital of the Russian Academy of Sciences.

### Patient selection

From 32 patients that underwent single-level myelopathy between December 2021 and June 2022, a total of 26 patients were enrolled, 13 of whom underwent EX assisted ACDF and the rest with OM. Patients were chosen in a randomized fashion. All had DCM presentations that were identified and confirmed by magnetic resonance imaging (MRI). All patients underwent single-level ACDF with the assistance of either the OM or EX. Patients with persistent myelopathic symptoms despite 6 weeks of conservative treatment were also included, in contrast, those with radiculopathy, tumors, infections, trauma, deformity, and a past history of cervical spine surgery were excluded.

### Study design

All the surgeries were performed by a senior neurosurgeon who was assisted by a resident. Operations in both groups were performed by the same surgeons to avoid bias. All the surgeons received 2 weeks of mandatory training in EX before using it in a real patient. Patients with degenerative disease planned for single-level ACDF using an EX were enrolled in a prospective cohort study. Similar age, sex and procedure-matched patients planned for OM-assisted ACDF served as the control group. We were granted ethical committee approval and Thai clinical trials registry number TCTR20221103003.

The patient demographic characteristics, radiological features, operative records, and complications were recorded. Postoperatively, the patients were assessed for new or worsened sensory or motor deficits and bladder bowel dysfunction.

Immediately at the end of each procedure, a survey was conducted where the participants had to answer a standardized questionnaire that included questions regarding the image quality of the EX, the illumination of the operative field, adjustment of magnification, focal length, depth perception, position and working angle of the EX, intraoperative handling of the instrument, convenience for the operating surgeon, assistant and scrub nurse, and the need to convert to OM during the surgery. Finally, we inquired if in the future, they would continue using the EX and recommend it to their colleagues. Postoperatively, the video recordings were analyzed to measure the operative time and evaluate the image quality when the EX or OM was used.

### Statistical analysis

Statistical analysis was implemented using SPSS 26.0 statistical software (SPSS, Inc., Chicago, IL, USA). Categorical variables were compared using Chi-square test. Continuous variables were expressed as mean and standard deviation and compared using *t*-test if normally distributed. The *p* value of <0.05 was considered statistically significant.

### Device properties

We used a previously described device (13), this EX consists of a 48 megapixels 4K 1,080p industrial video microscope camera with a 1X-130X C mount lens to a cantilever. The portable stand allows manual positioning of the camera. A ring light composed of 56 LED bulbs provides focused shadow-free illumination with adjustable brightness. All parts were sterilized with ethylene oxide. The surgical field was projected to a 55″ 2K television screen. We purchased the EX at a price of approximately US$ 150. The OPMI VARIO 700 (Carl Zeiss Meditec AG) was used for the conventional binocular OM-assisted groups. The cost was lowered from our previous work (2).

## Results

### General

A total of 26 subjects with DCM were included in this study, consisting of the levels C3–C4 (three cases), C4–C5 (11 cases), C5–C6 (11 cases) and C6–C7 (one case). In the totality of patients, the compression was successfully removed. The mean age was 51.6 ± 10.9 years (range of 34–71 years); The mean operation time was 113.8 ± 14.7 min (range of 88–138 min); The mean surgical blood loss was 30.4 ± 8.2 ml (range of 20–50 ml); The mean hospital stay was 3.2 ± 0.5 days (range of 3–5 days). The patient demographics, diagnosis, and operative data are shown in Table 1.

**Table 1:** Showing the patient demographics, diagnosis, and operative data.

Group	Age	Sex	Level	Blood loss (ml)	Operative time (min)	Hospital stay (days)	Complication
EX	44	M	C3–C4	30	126	4	No
	36	M	C3–C4	30	118	3	No
	38	M	C4–C5	40	108	3	No
	41	M	C4–C5	20	97	5	No
	53	F	C4–C5	40	107	4	No
	65	F	C4–C5	30	108	3	No
	60	F	C4–C5	20	104	3	No
	40	M	C4–C5	40	100	3	No
	55	F	C5–C6	20	92	3	No
	61	F	C5–C6	20	88	3	No
	63	M	C5–C6	30	111	3	No
	57	M	C5–C6	40	100	3	No
	40	F	C6–C7	30	136	4	No
OM	50	F	C3–C4	20	121	3	No
	41	F	C4–C5	30	129	3	No
	63	M	C4–C5	50	131	3	No
	46	M	C4–C5	40	107	3	No
	34	F	C4–C5	20	122	3	No
	38	M	C4–C5	30	100	3	No
	67	F	C5–C6	30	128	3	No
	45	M	C5–C6	30	136	3	No
	59	M	C5–C6	30	96	3	No
	54	F	C5–C6	40	108	3	No
	60	M	C5–C6	30	125	3	No
	71	F	C5–C6	20	138	4	No
	60	F	C5–C6	30	122	3	No
Mean	51.6			30.4	113.8	3.2	
SD	10.9			8.2	14.7	0.5	

Twenty-six operations were performed. Each surgery was performed by either two consultants or a consultant and a resident. There was no interruption during the procedure due to any technical problem. There was no conversion from the EX to the OM in any procedure. The operative time was less in the EX group when compared to the OM group although not statistically significant. The average intraoperative blood loss was significantly more in the OM group compared to the EX group (*p* = 0.021). The mean hospital stays were longer in the EX group compared to the OM group (3.38 ± 0.650 vs. 3.08 ± 0.277). No intraoperative complications occurred in either group. The comparison between the two groups is shown in Table 2.

**Table 2:** Comparison between the OM and EX groups.

Clinical evaluation			
Characteristic	EX group (*n* = 13)	OM group (*n* = 13)	*p* value
Sex, M/F	7/6	6/7	1.000
Involved level (C3–C4/C4–C5/C5–C6/C6–C7)	2/6/4/1	1/5/7/0	0.524
Mean age, years	50.23 ± 10.624	52.92 ± 11.572	0.542
Mean operative time, min	107.31 ± 13.332	120.23 ± 13.442	0.817
Mean intraoperative blood loss, ml	30 ± 8.165	30.77 ± 8.623	0.021
Hospital stay, days	3.38 ± 0.650	3.08 ± 0.277	0.130
Complications	0	0	

### Intraoperative setup

The overall intraoperative setup including patient positioning, surgical instruments and surgical technique of using the low-cost EX was essentially the same as the OM group.

The final setup of the patient and operating room for the EX was identical as the OM group. Time of preparation ranged between 4 and 6 min (mean of 5 min). Verification of the low-cost EX was done prior to starting the procedure by confirming the support arm movement, zoom of the camera and light intensity. As previously described (8) illumination optimal working distance is in the range of 40 and 60 cm. The quality of the image and surgeon's comfort were higher even when compared with surgical loupes, however, the lack of 3D imaging continues to be a negative factor.

Variable positioning of the camera can sometimes be a challenging consequence of the low versatility in the arm support movement, something that could be fixed by adapting another type of arm, however, spine surgery does not require significant movement as the camera remained in the same position most of the surgery.

The 32GB disc space allowed only 5 h of recording time, making it imperative to transfer files between surgeries. We found no intraoperative complications. The operating room team experience, including surgeon, assistant, nurses and anesthesiologist responded positively to the use of the low-cost EX. By everybody looking at the screen, superiority in teaching and feeling of inclusiveness in comparison with OM were the main consensus by the team.

### Survey

**Figure 1:**
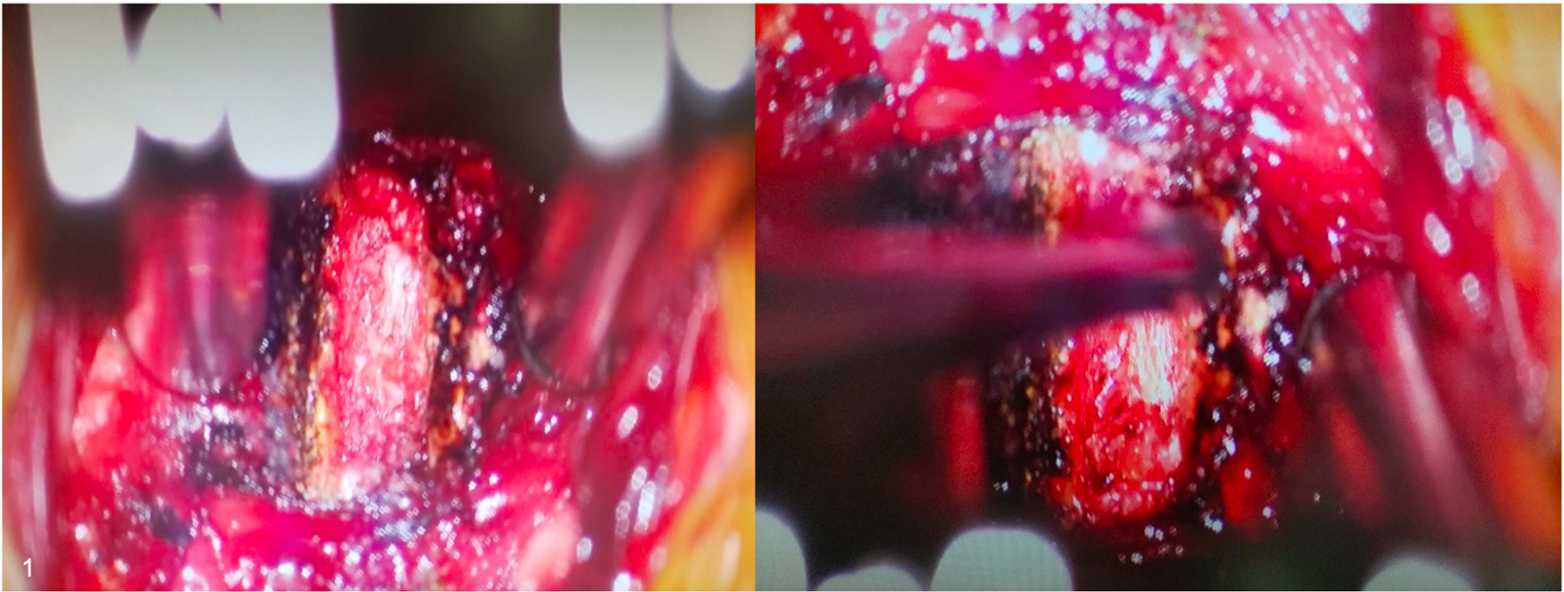
Intraoperative visualization of an ACDF procedure with the OM.

**Figure 2:**
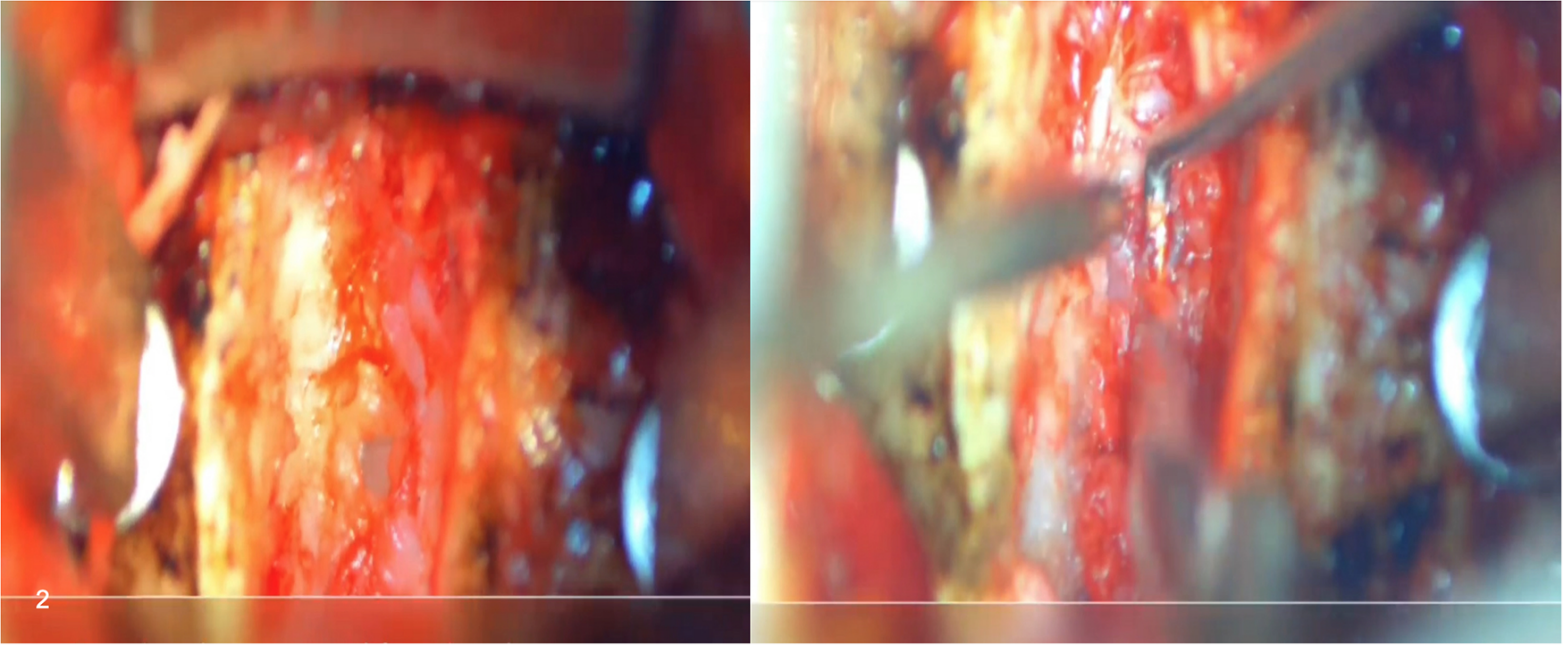
Intraoperative visualization of an ACDF procedure with the EX.

We interviewed 10 neurosurgeons. Of them, 60% were residents and the remaining consultants. Most (70%) of them found the brightness inferior to the conventional OM and the remaining 30% found the brightness similar to the OM. All agreed that the image quality of the surgical field was inferior to OM, nevertheless, zooming received a neutral response, we recommend taking into account the type of surgery before using the EX (Figures 1, 2).

About 70% of them felt that the ease of using the EX was similar to the OM. The same percentage of residents and scrub nurses strongly agreed that it was better for them as compared to a conventional OM, this was similar to the response to teaching, where 80% strongly agreed it was easier when using the EX. This follows previous reports of the main strength when compared with OM is that allows participation of every member in the operating room (14).

Almost all (90%) agreed that it was easier to set up the EX compared to OM preoperatively. The adjustment during surgery got a positive response, with almost 60% agreeing it was easier to adjust the position and angle of the EX than conventional OM.

None of the operating surgeons felt the need to switch to conventional OM during the procedure. All of them unanimously approved the low-cost EX and agreed to recommend or use it again.

## Discussion

OM has become an indispensable part of spine surgery since its introduction in 1977 (5, 6). It is used in the field of cranial and spinal surgery for better illumination, magnification of tissue and stereopsis. Because of the high costs associated with it, only a quarter of the world's population has access to microneurosurgical facilities (15). This has led to the development of newer visualization techniques causing a rapid evolution of exoscopes and endoscopes in the past decade. Industrial microscope cameras are a low-cost alternative to conventional surgical visualization systems in low and middle-income countries (LMICS) (8, 10, 16, 17).

In the present study, there was no conversion from the EX to the OM in any procedure. The mean operative time and intraoperative blood loss were less in the EX group compared to the OM group. The mean hospital stay was more in the EX group compared to the OM group. There were no surgical complications related to the use of the EX or OM. These results were similar to previously published reports on the use of EX systems in neurosurgery (5–7).

We were able to achieve superior operative results with our low-cost EX compared to the conventional binocular OM. This device has gained widespread popularity due to ergonomics and improvement in the surgeon's comfort (3, 5, 8, 11). The surgical scope that is visualized in any type of television screen permits a comfortable posture. A more favorable and less obstructive surgical field by not limiting the working distance results in an improved capacity of manipulation of the instruments (5, 8, 11). Increased awareness and better interaction with the rest of the team is found when projecting the surgery into a large screen and freeing the surgeon's neck. Several studies have shown that the team perceive a higher sense of usefulness and involvement when compared with binocular OM (8, 18). Standard sterilization and carriage from one place to another is simple due to the reduced size and weight of our device. This is consistent with other findings in the literature (9, 11).

Our study revealed some significant limitations of this system, including slightly inferior image quality and illumination compared with the conventional binocular OM. About 70% of the surgeons found the brightness inferior to the conventional OM and almost all of them agreed that the image quality of the surgical field was inferior to binocular OM. Our findings are consistent with previous literature showing that depth perception, quality of the image, and illumination of the EX were rated as inferior to the OM in ACDF procedures (5, 6). However, Oertel and Burkhardt, in their studies on 11 spinal and five cranial procedures using VITCOM 3D system (Karl Storz), reported an image quality that can be compared to that of the OM and advocated its use for less complex procedures (7).

According to our senior neurosurgeons users, the lack of stereopsis was a major drawback, substantiating previous findings in literature (3, 5, 6, 8, 11). Existing EX systems rely on 3D cameras to maintain image quality and depth perception while at the same time being ergonomically matchless compared to the OM. Novel technologies are equipped with 4K-3D displays, fluorescent filters for 5-aminolevulinic acid, indocyanine video-angiography and pneumatic arms (11, 19). The main restrictive factor remains the high cost of such systems. The cost of currently available surgical exoscopes range from US$250,000 to US$1,500,000 (8, 20). While refined robot-assisted movements and fluorescent filters may certainly assist in complex surgeries, most of the neurosurgical operations in LMICS are still performed with no magnification assistance (not counting surgical loupes) due to the lack of equipment (8, 15). We hope that the experiences with our $150 low-cost EX may inspire neurosurgeons in LMICs to seek similar solutions for performing microneurosurgery.

## Limitations of the study

The study aim was to evaluate our operative experience with an initial patient cohort. The size of the sample size makes statistical analysis not possible. We elected this specific surgical procedure because of the relative simplicity and familiarity for neurosurgeons worldwide. However, using the low-cost EX for surgeries with higher complexity needs further investigation. In addition, some bias could be present as the survey was filled by some authors that are included in the article. We believe that it is imperative to continue the investigation into the potential uses of the low-cost EX to determine its reliability and efficacy when compared to the OM. Larger cohort of patients could prove indispensable to establish this.

## Conclusion

Our study demonstrated that the low-cost EX it's a safe and effective alternative for OM-assisted ACDF with the benefit of great ergonomy, comfort and simplicity and furthermore serves as an essential tool for education and training purposes. Notwithstanding, our study revealed some crucial limitations of this technology, including slightly lower image quality and illumination compared with the conventional binocular OM. The low cost exoscope can be used as a surrogate for OM in spinal surgery to accomplish great surgical outcomes.

## Data Availability

The original contributions presented in the study are included in the article/Supplementary Material, further inquiries can be directed to the corresponding authors.
